# THE EFFECTIVENESS OF REIMAGINE AGING: AN INTERVENTION TO REDUCE INTERNALIZED AGEISM IN OLDER ADULTS

**DOI:** 10.1093/geroni/igad104.3759

**Published:** 2023-12-21

**Authors:** Dallas Murphy, Michelle Porter, Corey Mackenzie, Judith Chipperfield

**Affiliations:** University of Manitoba, Winnipeg, Manitoba, Canada; University of Manitoba, Winnipeg, Manitoba, Canada; University of Manitoba, Winnipeg, Manitoba, Canada; University of Manitoba, Winnipeg, Manitoba, Canada

## Abstract

A life time of exposure to ageist messaging and beliefs may lead older adults to internalize this messaging and direct it inwards, or towards their peers. This internalized ageism can have severe multi-faceted consequences on the well-being of older adults. However, little research has addressed reducing internalized ageism. Thus, Reimagine Aging, a six-week process-based intervention to reduce internalized ageism was designed and implemented, using tools of education, acceptance and commitment therapy, and attributional retraining to target theoretically based mechanisms of change. The data from 72 community-dwelling older adults (M = 70.4 years, SD = 6.4 years) were analyzed for changes in perceptions of aging and mechanisms of change. Participants completed questionnaires prior to the intervention, immediately following the intervention, and at a two-month follow-up. Results demonstrated that participants’ self-perceptions of aging (partial eta squared = 0.37, p < .001) and perceptions of older adults (partial eta squared = 0.27, p < .001) became significantly more positive and maintained these gains at follow-up, associated with large effect sizes. Furthermore, the increases in self-perceptions of aging were mediated by increases in the theory-related mechanisms of psychological flexibility, mindfulness, and perceived control. Positive gains in perceptions of older adults were mediated by these process-based factors to a lesser degree. This study provides initial support for the effectiveness of this process-based intervention targeting a reduction of internalized ageism. This has the potential to reduce the harmful consequences of internalized ageism impacting older adults globally.

